# Implementation of a Pilot Study to Enhance the Annual Wellness Visit for Older Adults With Cognitive Impairment

**DOI:** 10.1093/geroni/igaf122.1068

**Published:** 2025-12-31

**Authors:** Danny Scerpella, Danielle Peereboom, Jennifer Wolff

**Affiliations:** Johns Hopkins Bloomberg School of Public Health, Baltimore, Maryland, United States; Johns Hopkins Bloomberg School of Public Health, Baltimore, Maryland, United States; Johns Hopkins University - School of Public Health, Baltimore, Maryland, United States

## Abstract

The Medicare Annual Wellness Visit (AWV) is a routine visit for patients designed to monitor ongoing health status. The AWV requires detection of cognitive impairment through a health risk assessment that probes subjective memory concerns and “direct observation” during the visit. While the AWV holds promise for overcoming dementia care gaps, completion rates lag for older adults with diagnosed dementia. The goal of this pilot project is to generate a proof-of-concept for enhanced primary care workflows to increase the effectiveness of the AWV as a component of a dementia care planning pathway. This project focuses on nurse-led AWV and involves co-development of workflow enhancements including systematic cognitive screening protocols, expanded AWV education and follow-up, creation of patient priority dashboards (patients with diagnosed dementia, patients with a high dementia risk algorithm score, patients in the high acuity clinic) and implementation of a dementia risk algorithm for patients. In collaboration with clinical partners in leadership, staffing, and education, enhancements are identified and studied using PDSA (plan, do, study, act) cycle quality improvement methodology. We discuss the process for implementation within two clinics at a large primary care health organization, including: engaging administrative and clinic-level stakeholders and champions to enhance existing dementia-specific care pathways; liaising with clinicians and staff to identify priority patients; and generating and disseminating regular data tracking reports to inform adaptations. Lessons learned reinforce the value of study design that is co-developed, adaptable, and responsive to health system workflows and priorities.

